# A rare case of melioidosis presenting as myositis in Sri Lanka

**DOI:** 10.1186/s12879-022-07515-y

**Published:** 2022-06-15

**Authors:** Sanura Malinda Pallegoda Vithana, Liyanage Sashika Chathuranga, Saman Jayasinghe, Edippuli Arachchige Don Udayakumara

**Affiliations:** National Hospital Kandy, Kandy, Sri Lanka

**Keywords:** Melioidosis, *Burkholderia pseudomallei*, Myositis, Tropical disease, Rare presentation, Surgical debridement, Case report

## Abstract

**Background:**

Melioidosis caused by *Burkholderia pseudomallei* is an emerging infection in Sri Lanka with a high case fatality rate. The disease usually manifests as pneumonia, however multisystem involvement is common. Myositis is an extremely rare occurrence and this is the only documented case where the initial presentation of melioidosis has been myositis and later complicated to myonecrosis.

**Case presentation:**

A 45-year-old gentleman with pre-existing diabetes presented with a tender, right thigh lump for 1 week duration without any history of trauma or infection. Investigations revealed neutrophil leukocytosis, high erythrocyte sedimentation rate (ESR) and C-reactive protein (CRP) levels whilst ultrasonography showed focal myositis of right quadriceps. The patient went into sepsis amidst antibacterial treatment which warranted urgent surgery. At surgery, a large intramuscular abscess with myonecrosis was observed within vastus medialis which was completely drained and pus was taken for culture which eventually isolated *Burkholderia pseudomallei*. Melioidosis was diagnosed and intravenous meropenem was prescribed for 3 weeks. Following complete recovery, the patient was discharged on doxycycline and trimethoprim sulfamethoxazole for 3 months.

**Conclusions:**

Melioidosis, an endemic disease in south east Asia and northern Australia, is an emerging infection in Sri Lanka. Myositis is a rare presentation of the disease that can lead to myonecrosis and abscess formation which can cause rapid disease escalation and sepsis. Early surgical intervention may be life-saving in such cases where antibiotic therapy alone may not suffice.

## Background

Melioidosis often termed “the great imitator” is a disease endemic to south east Asia and northern Australia with a high case fatality rate. Although not endemic to Sri Lanka, increasing number of cases are being reported especially in the north western and central regions of the country [1]. It is caused by the Gram-negative bacterium, *Burkholderia pseudomallei *(*B. pseudomallei*) and is principally infected through percutaneous inoculation and by inhalation of aerosolized bacteria [2].

Diagnosis of melioidosis is difficult. It requires high clinical suspicion and laboratory tests to confirm. Bacterial culture, serological tests, polymerase chain reaction (PCR) based tests and more recently rapid specific identification technologies such as gene sequencing and matrix-assisted laser desorption ionization time-of-flight mass spectrometry (MALDI-TOF MS) are used [1, 3].

Melioidosis can have a vast array of presentations ranging from localized infection to disseminated infection and sepsis. Duration of illness also can vary from acute infection to chronic disease often mimicking tuberculosis and can even remain latent for many years [1, 2]. The commonest presentation of the disease is pneumonia, with more than half of all cases accounting for it [2]. Multiple abscesses involving liver, spleen, prostate and brain are common manifestations of the disease. Although soft tissue involvement is common, myositis is a rare manifestation [2]. Only one such occurrence has been reported in Sri Lanka [1].

## Case presentation

A 45-year-old truck driver from a village in Kurunegala district presented to surgical casualty with right thigh pain and swelling for 1 week. He denied trauma to the region or violent contraction of associated muscles. This had been associated with significant loss of appetite however fever and fatigue were absent. He was a diagnosed patient with diabetes on oral anti-hyperglycaemic medication and his control was poor (repeated fasting plasma glucose levels > 150 mg/dL during hospital stay). He was put on soluble insulin after consulting the endocrinology team which drastically improved the glycaemic control subsequently. On examination he was icteric but not pale and was hemodynamically stable (heart rate—84 beats per minute, blood pressure—110/70 mmHg, respiratory rate—14 breaths/min). There was an ill-defined anterolateral lump in the right thigh measuring 15 cm × 10 cm in size which was tender. No overlying erythema, warmth or crepitus was noticed and inguinal lymphadenopathy was absent. Apart from a mild hepatomegaly, rest of the systems examination including respiratory was normal.

His initial blood investigations (Table 1) revealed marked neutrophil leukocytosis, elevated erythrocyte sedimentation rate (ESR), C-reactive protein (CRP) and creatine phosphokinase (CPK) levels. Liver functions including prothrombin time/international normalized ratio (PT/INR) were deranged. Transaminases were only moderately elevated. Renal function tests and urine analysis were normal. Blood cultures were negative and blood picture revealed evidence of infection with mild thrombocytopenia. Ultrasonography of the lump was reported as focal myositis and abdominal ultrasound showed mild hepatomegaly with grade 1 fatty liver with no evidence of abscess formation. Magnetic resonance imaging (MRI) would have characterized the lesion better but was not considered owing to limited MRI facilities in our setting. Chest radiography was normal. Empiric antibiotic therapy with intravenous (IV) meropenem 1 g 8 hourly and clindamycin 600 mg 8 hourly was initiated.

**Table 1:** Blood investigations

Test	Value	Normal range
White blood cells (WBC)	30 × 10^9^/L	4–11 × 10^9^/L
Haemoglobin	12.4 g/dL	13.2–16.6 g/dL
Platelets	89 × 10^9^/L	150–450 × 10^9^/L
Aspartate transaminase (AST)	130 IU/L	10–40 IU/L
Alanine transaminase (ALT)	280 IU/L	7–56 IU/L
Total bilirubin	150 μmol/L	3–20 μmol/L
Direct bilirubin	88 μmol/L	0–14 μmol/L
Alkaline phosphatase (ALP)	670 IU/L	90–300 IU/L
Gamma glutamyl transferase (GGT)	213 IU/L	0–70 IU/L
PT/INR	1.84	< 1.1
S. creatinine	1.3 mg/dL	0.7–1.5 mg/dL
ESR	94 mm/h	< 10 mm/hr
CRP	321 mg/L	< 10 mg/L
CPK	281 IU/L	< 171 IU/L

The patient developed an unprovoked generalized tonic–clonic seizure on the 3rd day of hospital stay which spontaneously resolved without medication in 5 min. The timing of seizure did not coincide with drug administration. Subsequent computed tomography (CT) of the brain was normal. Blood glucose, serum electrolytes, acid–base status and blood gases were normal. Over the next 24 h, the patient gradually deteriorated with high fever (Temperature > 38 °C), persistent tachycardia of 110 beats per minute and a drop in Glasgow Coma Scale (GCS) to 13; his CRP was elevated to 360, serum lactate to 3.3 and had PT/INR of 1.6. He had gone into septic shock and acute liver failure with grade 2 hepatic encephalopathy. Liver failure regimen was implemented thereafter.

The subject was taken to the operating room and exploration of the thigh lump was undertaken as his clinical and biochemical markers of sepsis worsened irrespective of antibiotics. It revealed an abscess deep to sartorius muscle within the substance of vastus lateralis muscle with significant inflammation and myonecrosis. Pus was taken for culture and all necrotic tissues were removed and pus was drained. Thorough irrigation with saline was undertaken and the wound was packed with povidone iodine and kept open; IV antibiotics were continued. The patient had a drastic recovery following surgical debridement. Pus and tissue cultures were positive for *B. pseudomallei* which was susceptible to meropenem, trimethoprim sulfamethoxazole (TMP-SMX) and doxycycline (Table 2). A diagnosis of melioidosis was made and change in antibiotic regimen according to antibiotic susceptibility and current guidelines was implemented where meropenem 1 g 8 hourly was continued for 21 days and clindamycin was stopped. Doxycycline 200 mg daily and TMP-SMX 320 mg bd was added after 2 weeks of admission and was continued after discharge as eradication therapy for 3 months. His Human immunodeficiency virus (HIV) and hepatitis B, C screening was negative. After 3 weeks of in-hospital stay, the patient had a complete recovery and the wound was closed with sutures. He was discharged subsequently and followed up at clinic to date and currently symptom free.

**Table 2:** Antibiotic susceptibility pattern for *B. pseudomallei*

Antibiotic	Susceptibility
Ampicillin/amoxicillin	–
Penicillin	–
Cloxacillin	–
Cefotaxime	–
Clindamycin	–
Meropenem	Sensitive
TMP-SMX	Sensitive
Doxycycline	Sensitive
Erythromycin	–

## Discussion and conclusion

Melioidosis, a multisystemic disease with a variety of presentations has been virtually a novel disease until recently in the island nation of Sri Lanka. However, over the last decade this has changed with 250 culture positive cases being reported [4]. Most of the cases were reported from Western and North Western Provinces and very little from the central highlands. Our patient a driver by profession is based in the North Western province which is a hotspot for this emerging disease [4]. A review article on island wide melioidosis cases describes that 71.6% of all cases were male and many of them were farmers. An interesting fact is that 15% of all males affected were drivers which can be explained by their constant exposure to dust [4]. Aerosolized *Burkholderia *spp. in dust particles to which drivers in this region are constantly exposed, greatly increase the risk of inoculation and subsequent infection.

Sri Lankan data, similar to international studies have shown respiratory involvement to be predominant among cases (28%) [4]. A Sri Lankan study revealed 20% of cases with musculoskeletal involvement, however whether they involve only bone, muscle, or both were not mentioned [4]. An Australian study has reported the overall soft tissue involvement to be minimal; 19 out of 540 cases [2]. Myositis has only been reported once in a hospital less than five kilometers from Kandy National Hospital to which this patient was admitted. It describes a patient with myositis affecting the same group of muscles as in our case. However, the patient had an initial presentation of pneumonia and had undergone the treatment [1]. In addition to myositis, our subject had myonecrosis and an intramuscular abscess within quadriceps. Ours is the only case reported with myositis being the initial presentation of melioidosis and the first to have undergone successful surgical debridement for melioidosis myositis.

Liver and splenic abscesses are a common presentation of the disease especially in Thailand and most have been known to resolve with prolonged antibiotic therapy alone [2]. Even though liver functions were deranged, no evidence of liver abscess was found on imaging in our subject. Acute liver injury could therefore be attributed to sepsis in this case. Rapid normalization of liver functions following medical and surgical management to counter sepsis further supports this. Neurological manifestations of melioidosis include brain abscess, meningoencephalitis, transverse myelitis and status epilepticus [4]. Recent animal studies have shown the entry of *B. pseudomallei* from nasal mucosa via olfactory nerve to the brain [2]. The single seizure episode in our patient most likely would have been due to sepsis and/or hepatic encephalopathy following acute liver injury. Another probable cause is meropenem which even though rare can cause seizures as an adverse effect especially in the elderly. This however is quite unlikely in this patient considering the single episode and timing of the seizure. Other causes of seizures such as electrolyte imbalances, acid–base disturbances and intra cranial lesions were excluded. However, brain involvement without evidence of brain abscess caused by *Burkholderia *spp. cannot be completely disregarded and such presentations may require further investigations including cerebro-spinal fluid (CSF) analysis and MRI.

Diabetes mellitus (DM) has been documented as an important risk factor in disease acquisition in many studies including the “20-year prospective Darwin study on melioidosis” [2, 5]. According to another study encompassing global data, it was found that up to 60% with the disease were having DM, type 2 being predominant [5]. One study involving a mouse model has shown the importance of neutrophil function in resisting melioidosis infection [6]. Neutrophil dysfunction seen in DM therefore weakens immunity and increases the chance of infection which is reflected by the high percentile of those affected being diabetic. In addition to have played a major role in disease acquisition, DM would have greatly contributed to the sudden escalation of sepsis seen in the subject owing to immunological dysfunction.

Management of melioidosis encompasses an initial intensive therapy followed by eradication regimen. Antibiotic options for intensive therapy include ceftazidime, meropenem and TMP-SMX [5, 7]. Intensive therapy for 10–14 days is recommended for isolated cutaneous or pulmonary disease whereas for severe deep-seated infection, bone or central nervous system involvement, 4 to 8 weeks of IV therapy may be warranted [7]. Antibiotic options for eradication include TMP-SMX, doxycycline, chloramphenicol and co-amoxiclav (as a third line agent in case of treatment failure or relapse) [7, 8]. Eradication therapy needs to be continued for 3 to 6 months guided by clinical and biochemical response (WBC, CRP) [7]. According to the antibiotic sensitivity pattern and local microbiology guidelines, the patient was prescribed IV meropenem for 21 days followed by TMP-SMX and doxycycline as eradication treatment on discharge. Prolonged IV therapy was not recommended even though he had one episode of seizure; the reason for which could have been multifold rather than infection. In case of post exposure prophylaxis, current recommendations are TMP-SMX or co amoxiclav for a duration of 21 days whilst keeping an eye for any evidence of infection [7]. This was not required for the ward or lab staff in our case. In addition to medical management, ours is a rare case where surgical drainage and debridement was undertaken for melioidosis myositis. In fact, the condition did not improve amidst use of antibiotics until surgical debridement was undertaken emphasizing the importance of early intervention in the presence of abscess formation and myonecrosis.

Melioidosis can present as primary myositis and thus needs to be considered as an important differential diagnosis in those presenting with localized muscle pain and soft tissue swelling in endemic areas. Melioidosis myositis can lead to myonecrosis and abscess formation which can result in rapid disease escalation and sepsis. It is therefore essential to have a high degree of suspicion and carry out early diagnostic imaging followed by early surgical intervention to prevent disease progression and mortality.

## Data Availability

Not applicable.

## Abbreviations

ALP: Alkaline phosphatase
ALT: Alanine transaminase
AST: Aspartate transaminase
CPK: Creatine phosphokinase
CRP: C-reactive protein
CT: Computerized tomography
DM: Diabetes mellitus
ESR: Erythrocyte sedimentation rate
GCS: Glasgow Coma Scale
GGT: Gamma glutamyl transferase
HIV: Human immunodeficiency virus
IV: Intra-venous
MALDI-TOF MS: Matrix-assisted laser desorption ionization time-of-flight mass spectrometry
MRI: Magnetic resonance imaging
PCR: Polymerase chain reaction
PT INR: Prothrombin time international normalized ratio
TMP-SMX: Trimethoprim sulfamethoxazole
WBC: White blood cells
